# Skin in the game: a review of computational models of the skin

**DOI:** 10.1186/s13040-025-00471-8

**Published:** 2025-08-19

**Authors:** Seda Ceylan, Didem Demir, Cayla Harris, Semih Latif İpek, Vasileios Vavourakis, Marco Manca, Sandrine Dubrac, Roman Bauer

**Affiliations:** 1 Department of Bioengineering, Adana Alparslan Türkeş Science and Technology University, Adana, Türkiye; 2 Department of Chemistry and Chemical Process Technologies, Tarsus University, Mersin, Türkiye; 3 NICE research group, School of Computer Science and Electronic Engineering, University of Surrey, Guildford, United Kingdom; 4 Department of Food Engineering, Adana Alparslan Türkeş Science and Technology University, Adana, Türkiye; 5 Department of Chemistry, Kütahya Dumlupınar University, Kütahya, Türkiye; 6 Department of Medical Physics and Biomedical Engineering, University College London, London, UK; 7 In Silico Modelling Group, Department of Mechanical and Manufacturing Engineering, University of Cyprus, Nicosia, Cyprus; 8 SCimPulse Foundation, Geleen, The Netherlands; 9 Department of Dermatology, Venereology and Allergology, Medical University of Innsbruck, Innsbruck, Austria

**Keywords:** Skin, Dermis, Epidermis, Modelling, Skin biomechanics, Computational dermatology, In silico

## Abstract

With the vast advances in computing technology, computational (or in silico) modelling has emerged as a transformative tool in dermatology. These findings can provide novel insights into complex biological processes and aid in the development of innovative therapeutic and regenerative strategies for the skin. Modelling combines experimental data and knowledge across multiple disciplines, serving as a common framework to elucidate the workings of the skin. From a biomedical perspective, the mechanisms of skin diseases can be studied by simulating cellular interactions and signalling pathways. Computational investigations of these mechanisms can be categorised into two distinct approaches: data-driven and model-based. Data-driven approaches allow the diagnosis of skin diseases on the basis of data collection via imaging or feedback from portable sensors, often yielding performance exceeding that of their human counterparts. Model-based methods are well suited to address topics such as skin cell biology and biomechanics, contributing to wound healing and skin cancer research. Furthermore, such modelling has found utility in the development of virtual skin models and skin-on-chip devices, enabling the prediction of skin responses to various substances, including cosmetics and drugs. In the realm of dermatological surgery, computational tools have been instrumental in optimizing surgical planning and improving clinical outcomes. While significant advancements have been made, challenges such as data availability, model validation, and interdisciplinary collaboration persist. This review highlights the current state-of-the-art in computational modeling in dermatology, identifies key challenges, and outlines its prospects.

## The skin: a complex organ

The skin, the largest organ of the body, is a multilayered barrier that provides both physical and immunological protection. It plays a central role in numerous aspects of essential physiological function, including protection from germs and UV radiation, and the regulation of body temperature. These essential roles make it the subject of extensive biomedical and pharmaceutical research.

**Fig. 1:**
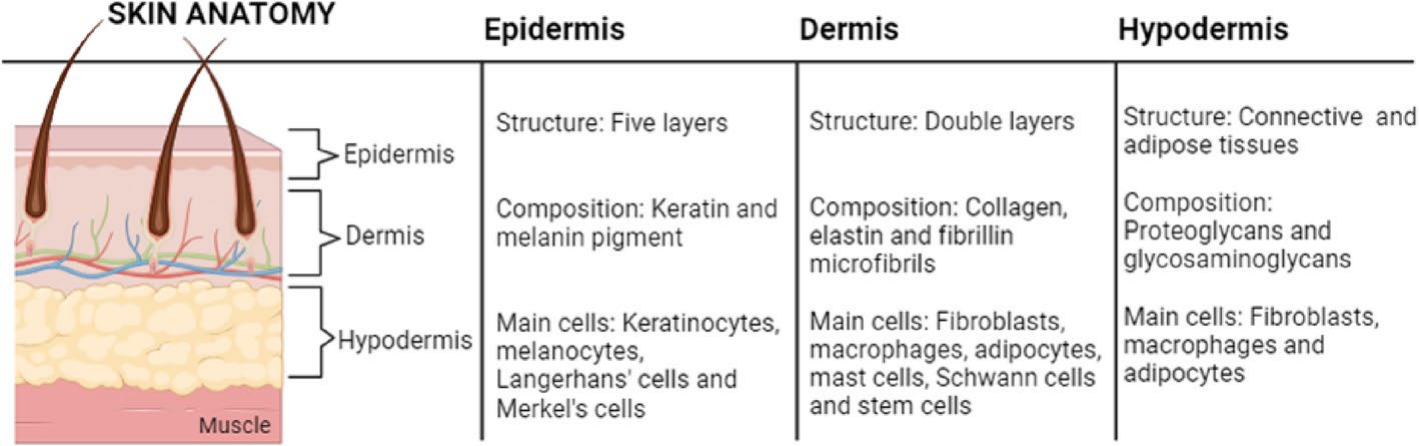
Anatomical skin layers and the basic content of each layer in terms of structure, composition and cells present

The multiple layers of this complex organ include the epidermis, the dermis, and the hypodermis (subcutaneous tissue) (Fig. 1). The epidermis is the outermost layer of the skin, providing the first protective barrier, and consists of four to five distinct layers: the *stratum basale*, *stratum spinosum*, *stratum granulosum*, *stratum lucidum* (only in soles and palms), and *stratum corneum*. Keratinocytes, melanocytes, Langerhans cells, and Merkel cells constitute the epidermis [1]. The dermis consists of two distinct areas: the superficial papillary dermis and the deeper reticular dermis. These areas contain collagen, mostly type I or type III, and elastic fibres such as elastin and fibrillin microfibrils [2]. Various cell types, including fibroblasts, immune cells (e.g., macrophages, mast cells), adipocytes and Schwann cells, populate the dermis, which also includes skin appendages such as hair follicles, sweat glands (eccrine), and apocrine and sebaceous glands [3]. The hypodermis is composed of highly-vascularized, loose, areolar connective tissue, consisting of adipocytes, fibroblasts and macrophages, embedded in an extracellular matrix rich in molecules such as proteoglycans and glycosaminoglycans [2] (Fig. 1).

Given the range and impact of skin diseases around the world, research is essential to advance the understanding of skin pathophysiology and to develop new treatments.

Skin diseases are recognized as important public health problems affecting millions of people, involving high costs for individuals and societies, and are among the leading causes of the global burden of disease. To date, more than 3,000 skin diseases, both acute and chronic, affecting individuals of all ages, sexes, ethnicities and social conditions have been described. Among these diseases, some can cause a significant deterioration in quality of life and represent substantial costs for patients [4].

Skin diseases encompass a spectrum of conditions, such as [5]:

- **Inflammation**: acne, dermatitis, eczema, hives, pityriasis rosea, and psoriasis
- **Viral infections**: herpes simplex, herpes zoster, and warts
- **Bacterial infections**: impetigo, folliculitis, cellulitis, and Lyme disease
- **Fungal infections**: athlete’s foot, jock itch, and ringworm
- **Pigmentation disorders**: age spots, freckles, melasma, and vitiligo
- **Tumors and cancers**: basal cell carcinoma, squamous cell carcinoma, and malignant melanoma
- **Other conditions**: traumatic injuries, rosacea, spider veins, and varicose veins

Among these, dermatitis, psoriasis, and melanoma are associated with some of the highest global values for Disability-Adjusted Life Years (DALYs) [6].

Research and development for the treatment of skin diseases worldwide is considerable. Given the extensive financial and temporal investments required for the development and introduction of a new drug to the market, combined with the long periods between preclinical testing and drug approval, the use of accurate in vitro models in early stages of drug development is becoming increasingly common [6]. A prerequisite to drug development is a deep understanding of pathogenic processes that help identify key targets in the skin. Mathematical modelling of skin diseases can help provide tools for understanding complex and dynamic pathological mechanisms. The power of mathematical models, coupled with in vitro experiments, can reveal previously unidentified regulatory mechanisms [7].

**Fig. 2:**
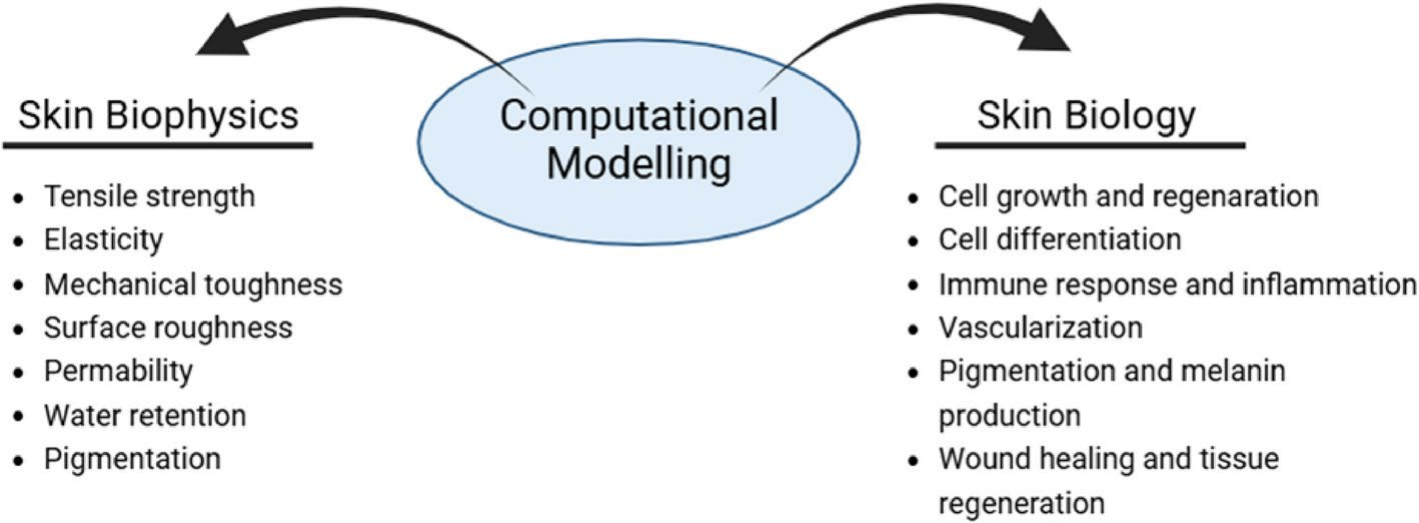
Computational model perspective for skin problems summarized by illustrating the main biological and physical properties of skin

Computational modelling, which encompasses mathematical modelling, is a discipline that aims to simulate complex systems by employing mathematics, physics, and computer science. A computational model contains a multitude of variables that define the characteristics of a system under study. Simulations are conducted by adjusting the variables, individually or in combination, and observing the outcomes. This enables scientists to perform thousands of simulated experiments in a very short time. The results of these simulated experiments can then be used to assess and compare both existing and novel hypotheses. Computational models can aid in the study of a biological system at multiple levels and across different scales. These include models of disease aetiology, such as molecular processes, cell–cell interactions, and changes affecting tissues and organs. This review summarises the current techniques of computational modelling dedicated to studying the skin, with applications in research into fundamental skin properties, diseases, healing, and ageing. We aim to define the current technologies already available and reveal the direction in which research and development is heading. This presents a compilation of studies on modelling the biological factors and physical structure of skin tissue, all of which play an active role in skin disease modelling studies. Accurate modelling of these diverse biological and physical properties of the skin is highly important for understanding the aetiology of skin diseases, developing treatment strategies, and testing new treatment methods. Examples of these properties are described in Fig. 2, which provides a schematic representation of the physical and biological properties that are frequently employed in the context of skin modelling studies. In summary, the success of research on skin diseases is based on accurate modelling of the complex biological and physical structure of the skin, which will be explored and evaluated within the scope of this review.

## Data-driven vs. model-based computational models

Fundamentally, there are two main types of approaches for computational modelling. The traditional type denotes those that are primarily *model-based*, relying on established theoretical frameworks and mathematical equations to represent the underlying phenomena. Clearly, these require some prior insight and knowledge of the complex system at hand. In the case of the skin, the model could incorporate equations describing diffusion in different layers of the skin, the fact that there are different layers and cell types, or some assumptions underlying collagen dynamics. Hence, while these models provide valuable insights, they can be challenging to conceive, as they often require significant domain expertise and can be limited by the accuracy of the underlying assumptions.

In contrast, *data-driven* models have emerged as powerful alternatives, particularly with the proliferation of large datasets and advancements in computing power. These models learn patterns directly from data without explicit theoretical assumptions. They excel at capturing complex relationships and making predictions in scenarios where the underlying mechanisms are poorly understood. For example, in the context of skin research, automated image analysis methods for the diagnosis of melanoma have been developed and successfully employed in clinical practice, as explained below.

Both model-based and data-driven approaches have advantages and disadvantages (Table 1), and the modeller can choose the one that leverages existing data and background knowledge to achieve the given goals of the research [9]. The availability of suitable hardware and software should also be considered. Different computational approaches can fit better with different resources. For example, mechanistic models can often be more computationally demanding since they are designed to capture the actual underlying mechanisms. In contrast, data-driven models are usually agnostic to the specific object of the research, and significant optimization has often been conducted to leverage these rather generic models for various applications. For example, artificial neural networks (ANNs) have gained significant attention because of their ability to model a wide range of data, including images, languages or videos. Along those lines, various open-source machine-learning (ML) and deep-learning (DL) libraries exist that allow the utilisation of a wide range of computer hardware [10]. Analogously, mechanistic simulators, such as in agent-based modelling (ABM) [11], are available to simulate skin biomechanics and biology. These methods often enable highly efficient simulations [12, 13]. Notably, a given computational model can also be hybrid, leveraging advantages of both approaches for different model components.

**Table 1:** Model-based vs. data-driven approaches

Category	Model-based (MB)	Data-driven (DD)
**Advantages**	-Provides explainable results based on established science	-Can discover patterns that may not have been identified before
	-Useful for hypothesis testing and understanding mechanisms	-Can analyse large, complex datasets effectively
	-Relatively data- and compute-efficient	-Adaptive to new data, enabling continual improvement
	-Generally, more straightforward to validate and gain regulatory approval due to transparency	-Highly effective for predictive analytics and diagnostics
	-Scalability allows more granular and accurate representation of complex systems	-Fast computations or/and low operational cost once model trained
**Disadvantages**	-Model accuracy: Based on assumptions that may reduce performance in benchmark comparisons	-Often lacks interpretability, rendering it a “black box” approach
	-Less adaptable to new, unforeseen patterns of data	-Demand for large, high-quality datasets
	-Limited by the accuracy of underlying theoretical models, verification is challenging	-Susceptible to biases present in the training data
	-Often requires significant domain expertise and dedicated training of user	-High computational resource requirements, especially during model training
**Data types**	-Experimentally and specifically measured variables (e.g., concentrations, rates, biomechanical measures)	-Large and multi-dimensional (e.g., images, time series, omics, sensor data)
	-Structured, parameter-focused	-Often unstructured or semi-structured. Labelled or semi-labelled
**Collection instruments and data acquisition**	-Lab equipment, assays, physiological sensors	-Wearables, scanners, photo/video cameras
	-In vitro/in vivo experiments, controlled experiments, theoretical analysis	-Real-world environments, observational studies, clinical trials
**Analysis & processing methods**	-Differential equations, system dynamics, rule-based models, agent-based modelling, finite element method, molecular dynamics simulation	-Machine learning, AI, statistical inference, data mining
	-Solvers of ordinary/partial differential equations (ODE/PDE) solvers, simulation software	-Neural networks, clustering, regression, principal-component analysis (PCA)
	-Experimental validation, sensitivity analysis	-Cross-validation, prediction accuracy

This table lists advantages, disadvantages and other aspects of these approaches. Please note that this comparison is not exhaustive

When domain experts understand the capabilities and utilisation of computational modelling and simulations, it becomes possible to test different hypotheses. Multiple approaches are usually required for a certain complex system to be understood, given that one never has access to full information at all levels. Hence, there are often gaps in our understanding that render various hypotheses possible. Using computational modelling, one can implement and compare such hypotheses and assess their consequences. In this way, it becomes possible to generate predictions that could then be experimentally verified to identify the most suitable hypothesis.

## Computational modelling in systems biology

Biological systems are often relatively complex; thus, to gain insights into how they function, it is crucial to obtain experimental information from different data modalities, such as omics, imaging, or biomechanical data. The availability of powerful and precise technologies to extract such data has enabled the generation of large and detailed sets of information at different spatial and temporal levels. Given the associated complexities, it is necessary to employ advanced quantitative methodologies to capture and make sense of these data. In addition to mathematical and analytical methods, computational approaches have gained significant attraction in the biological and medical communities, boosted by the accelerating performance gains of modern computer hardware and software. Notably, many computational models address open questions in the context of highly complex systems and multidisciplinary settings and thus foster the exchange of ideas across traditional fields.

**Fig. 3:**
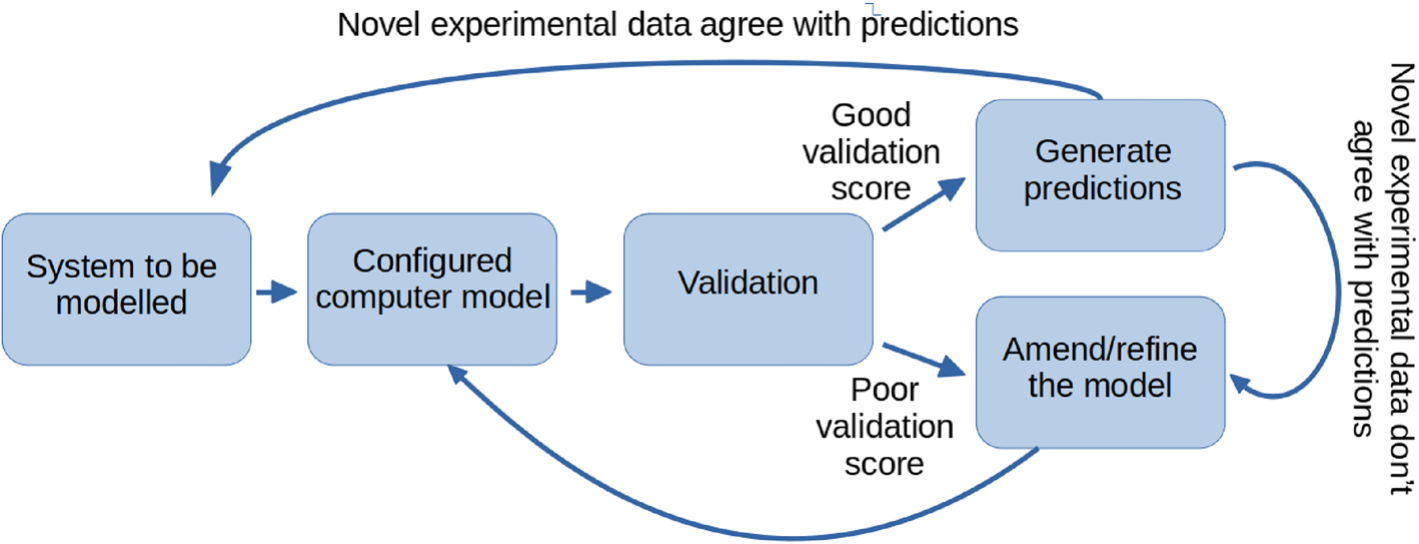
The approach of computational modelling in computational biology research (adapted from [8]). A given biological system, such as the skin, is modelled on the basis of an initial set of model parameters. Conditional upon satisfactory validation, novel and experimentally verifiable predictions can be generated and compared with new experimental data. If successful, a more detailed and comprehensive computational model can be devised in an iterative manner

From a conceptual perspective, computational modelling can be understood as a generic approach to simplify and formulate a model with predictive power. It is an iterative approach, as the level of detail and depth of the model can be refined in close interaction with experimental research. It is through this interaction that it becomes possible to make sense of highly complex datasets, which otherwise would surpass the abilities of the human mind. Figure 3 visualizes the computational approach in this iterative fashion.

The starting point of the circular approach is the selection of the system to be modelled. For instance, this type of system could be a cancerous skin sample. The next step is to create a computer model of the system, which requires certain assumptions and simplifications. The model is subsequently evaluated on a validation dataset that must be new— i.e., no information from the validation dataset can be used in the creation of the model. Depending on the validation results (such as accuracy of cancer diagnosis), the model must be revised and improved. This can be achieved by amending the initial assumptions and increasing the level of detail of the model. If the validation is satisfactory, novel hypotheses should be generated. Experimental verification will then provide information on whether the model needs revision or whether the system can be studied at greater depth.

Computational modelling is a generic methodology that aims to produce an idealized representation (such as using a mechanistic or data-driven approach, see Data-driven vs. model-based computational models section) of a complex system via continuous improvement through tight interactions with the experimental work of the system under consideration. Skin research can help address various research questions, such as those concerning skin cancer diagnosis. Notably, computer models often also generate experimentally verifiable predictions that allow assessment of the accuracy and realism of the model. Moreover, they can reduce the need for experimental research (including the use of animals and other ethically challenging methods) [8]. Despite the impressive capabilities and insights that computational methods have delivered in biomedicine, challenges in bridging gaps between wet-lab research and computational modelling remain present. Along those lines, computer scientists may lack expertise in biological and medical domains. On the other hand, it is often challenging for researchers without a strong computer science background to make use of custom research software. We address this gap here by providing a general overview of work in dermatology.

Given the extensive availability of software to employ computational modelling for a wide range of tasks, it is not surprising that a plethora of applications in systems biology have been addressed. Because of the central role that the skin plays in human physiology and well-being, extensive computational research has focused on understanding its role in health and disease. While it is beyond the scope of this review to describe this research comprehensively, we categorize and provide an overview of the most prevalent categories in the following sections.

## Computational models in skin research and applications

### Characterization of skin biophysics

One of the main purposes of computational modelling is to gain insights into the workings of complex systems. Computer models can bringing together existing knowledge and establish basic dynamics of the underlying processes. Moreover, they can be used to test and compare different hypotheses. In clinical investigations, diagnosis, and numerical modelling, understanding the mechanical characteristics of soft tissues and organs is crucial, which highlights the importance of exploring these attributes in the context of skin. In dermatology, significant fundamental research has been conducted via computational modelling. The review by Limbert [14] summarizes a number of seminal works employing a spectrum of different computational and mathematical techniques to model skin biophysics. This work presents various model-based approaches that consider detailed biophysical laws. However, current developments in AI techniques will likely play an increasingly important role in fundamental skin models. Limbert’s review investigates such data-driven methods that help generate predictions that can then be used to optimize therapeutic interventions. However, as in many other biomedical fields, data availability is a significant bottleneck, so interdisciplinary initiatives are needed to inform and improve relevant computational models. Mechanical qualities vary from person to person, and they change with time and under various circumstances. Gaining knowledge of these material properties adds to what can be included in computer simulations and organ prediction diagnostics. Characterizing the mechanical behaviour of skin and understanding the underlying mechanisms of deformation at different spatial scales is essential in a broad spectrum of applications, such as surgery, cosmetics, forensics, biomimetics, and engineering of protective gear or artificial grafts [18]. Therefore, accurate methods that can measure the mechanical characteristics of skin tissue are needed.

In Dwivedi et al. [20], an innovative approach was proposed to determine the mass density and elasticity of soft tissues by analysing their mechanical vibration response when stimulated externally by sound waves or a mechanical indenter. Soft sheets are subjected to a vibration test to gauge how they react to a continuous range of stimulation frequencies. Using 3D digital image correlation (DIC) and two high-speed cameras, frequency responses are gathered. To analytically simulate the free vibrations of continuous layered human skin and neoprene artificial sheet samples, an effective theoretical model was constructed. Finite element (FE) modelling, as well as comparisons with DIC measurements and frequency tests, demonstrated the excellent validity and correctness of the analytical simulations. This analytical approach was converted into a numerical program and was shown to reproduce the experimental measurements in the simulation. This study effectively demonstrates how experimental and in silico methods can be combined to generate highly realistic skin biophysics models. Nevertheless, how different skin properties (e.g., across age and skin types) can be measured in a non-invasive manner and modelled remains to be investigated.

Changes in the mechanical properties of the skin can be indicators of disease, highlighting the potential that computational models of skin biomechanics have in global health. A significant example is how elevated blood glucose levels associated with type 2 diabetes mellitus (T2DM) alters the skin’s mechanical characteristics. Bermudez et al. [19] compared the biomechanics of maximum stress load capabilities and elastic modulus to be significantly lower in murine skin affected by diabetes mellitus than in healthy individuals. Dwivedi et al. [20] also observed higher levels of anisotropy, meaning higher degrees of parallelism between collagen fibres, as well as general collagen loss in the skin of mice with T2DM, contributing to the observation in a reduction of skin stiffness.

Understanding the unique mechanical properties of skin is not only important in their response to health and disease, but also for engineering tools and technology that has the intention to work with these properties. Printable or flexible sensors are a field of medical innovation with groundbreaking potential, specifically in the realm of skin biophysics, where monitoring devices can be adapted to the unique, biological terrain by the specific design. Implementing medical technology into an adaptable medium that can be worn or integrated onto a physical body allows for easier and more compatible health monitoring.

Zhu et al. [82] created a real-time toolpath adaptation system for in situ 3D printing by estimating the motion and deformation of the target surface. A hydrogel-based sensor was printed via this printing technology on a porcine lung that had been deformed by breathing. Through electrical impedance tomography, a non-invasive method of measuring cardiac output and pulmonary artery pressure via a radiation-free medical imaging approach [83], the sensor continuously offers spatial mapping of deformation while adhering to the tissue surface. By building a training dataset of point clouds with pointwise correspondence among data samples on the basis of 3D scans, they were able to determine the linear shape basis model of the surface deformation via a machine learning method. Owing to its additive manufacturing capabilities, adaptive 3D printing technology of this type may improve robot-assisted medical treatments by enabling autonomous and direct printing of biological materials and wearable electronics on and within the human body.

Similar methods can be used in the context of 3D printing these biosensors directly onto the skin for various monitoring approaches. Internal temperature monitoring is fundamental in a range of health and disease, whether they identify localized temperature shifts that may indicate issues with inflammation or circulation, or more generalised shifts associated with thermoregulation or infection. Current work is focused on incorporating conductive polymers into printable mediums. Wei et al. [84] implemented these polymers into biocompatible and mechanically robust microneedles that can penetrate into the skin with a depth, diameter and angle optimised in their studies. Hwang et al. [85] created a skin-conformal temperature sensor array less than 2$\mu$m thick, allowing for comfort and mechanically appropriate deformation of the arrays when attached to skin.

Strong knowledge in skin biophysics is crucial for understanding the modalities of drugs and other chemical penetration into the tissue, which influences their toxicity and treatment efficacy. In this context, computational modelling can help increase the cost-effectiveness of drug delivery or assess the risk of various exposure types. Drug information is frequently found within clinical narratives, and drug regimens are subject to frequent changes due to factors such as intolerance or insurance-related issues, which complicates accurate modelling. In a research study, scientists created an informatics framework that uses natural language processing and ML techniques to determine patient drug exposure histories from electronic medical records [22]. In line with this, several studies have employed computational modelling to study the uptake of various molecules in the skin.

### The diseased and damaged skin

Skin pathology is unique in that it is directly observable via visual assessment. However, current diagnostic procedures still require assessment by formal medical personnel. This requirement for direct assessment by trained medical professionals is a common barrier in medicine in terms of accessibility, cost and resource availability, which is particularly problematic in remote or poorer regions of the world. Given the ease of observing the skin, skin diseases are ideal candidates for incorporating machine learning tools into modern medical diagnostics to improve accessibility and reduce the workload on the current medical workforce.

There has been a recent surge of interest in the use of images of healthy and diseased skin under different conditions to train AI models for diagnostic potential. As often observed when training different models, a significant bottleneck for this approach is the availability of training data. Pattern recognition algorithms require vast amounts of data demonstrating images that are classified as healthy or diseased. Owing to the potential demand, there are few complete datasets that possess the extent of information required to train a model for skin disease and damage classification.

A database for 1,000 research participants, 200 healthy volunteers and 800 dermatology patients with facial skin concerns was constructed for the study [80]. To categorize patients according to their skin conditions, an expert method has been devised (majority class). Here, an ANN that had been pretrained was chosen to construct the expert system. Since the dataset was not balanced, i.e., the number of samples varied significantly across skin conditions, a modified learning algorithm was employed. This algorithm treats the minority class as noise or interference during the training phase. Although the ANN achieved a high degree of accuracy, more assessment parameters suggest that it could be enhanced to more accurately predict the results. More sensitive and accurate findings may be obtained by increasing the number of factors and the number of patients or healthy volunteers, which will ultimately result in an effective classifier [80]. Notably, the number of research participants was significant and could be further expanded in number to improve the expert method in future studies. Overall, his study highlights the potential of using ANNs for effective skin diagnostics.

CNN algorithms can be used to extract higher- and higher-level representations of image content and are thus used for image classification; in dermatology, they extend towards the classification of skin lesions and the identification of whole skin types. Photographs of normal, oily, and dry skin were included in the collection; there were 112 photographs of normal skin, 120 images of oily skin, and 97 images of dry skin. A total of 1,316 pictures were obtained after the Contrast Limited Adaptive Histogram Equalisation (CLAHE) approach was used to improve image quality, and data augmentation by rotation was used to increase dataset variation. The accuracy of the model was 89.70% when tested on unknown data and verified via cross-validations [2].

An excellent study by Shah et al. [35] explored the role of machine learning techniques, such as ANNs and convolutional neural networks (CNNs), in identifying skin cancer. These methods are inspired by biological neural networks, where information is processed between a point of input and output. In this context, computational neural networks can take in the information provided through images of the skin, process and recognize patterns about this information, and provide an output as a classification of the condition of the skin. Shah et al. explored currently available ANN methods, where the network is made up of multiple layers of nodes, allowing different weights of importance assigned to each input. The added layers constitute deep learning, and combined with different weights of information being relayed at each layer, they provide a network that processes the images and identifies patterns in the texture and boundaries of the provided dataset of skin samples.

Some ANN techniques incorporate additional information, such as colour, symmetry, patient background and history as parameters. CNNs expand on this concept while distributing their weights between nodes evenly to allow for a reduction in parameters and a lower computational cost. This allows for higher efficiency in the image processing steps, such as hair and colour removal, and improved extraction of topological features that either deviate from healthy skin. The efficacy and performance of ANNs and CNNs in the early and efficient diagnosis of skin cancer are determined by evaluating studies on the detection of the disease. The findings of this study, which showed the potential for these technologies to increase accuracy in skin cancer diagnosis, showed that ANNs and CNNs were effective in early skin cancer identification when various datasets and hybrid models were used, as well as the ability of CNNs to discriminate between benign and malignant skin malignancies. This novel application of deep learning techniques for skin cancer detection may be key in saving time and effort in future diagnostic processes and highlights the vital need for automated systems for skin lesion recognition [35], with automation of skin cancer diagnosis procedures and the application of CNNs yielding more accurate categorization [36].

The identification of pathological skin markers via dermoscopy is laborious, especially in healthcare settings with limited resources [37]. Recent developments in computer vision [37] have helped overcome these limitations through the development of numerous smartphone-based apps or smartphone images [38] for early skin disease prediction. In a recent study, they processed over 45,000 high-quality photos from the HAM10000 dataset, which covers seven types of skin disorders, via a collection of sixteen unique DL-based CNN models. With the use of lesion photos, their algorithms were able to accurately diagnose and categorize skin conditions.

As it has been demonstrated by research on grokking (a delayed generalization occurring long after achieving near zero training error) [39], CNNs always benefit from larger dataset, as long as the computational infrastructure supports an arbitrarily deep architecture. It is, however, common in biomedical sciences to operate in settings where data resources are fragmented and access is limited. Thus, a valuable question to answer concerns what the minimum dataset size would be to effectively train a CNN. The answer is not straightforward, as the convergence of the testing and training errors is heavily affected by the task and the data distributions, and it can differ based on the network architecture (e.g. WideResNet [40], Xception [41], Pyramidal [42], etc.) and trade-offs based on the computational infrastructure. There are techniques to enhance learning performance when faced with data scarcity. When computational infrastructure is not the limiting factor, the most promising one is transfer learning [43, 44], a technique where data extraneous to the original task but similar in nature are introduced in early learning stages to learn general features, which are in later training steps fine-tuned on the smaller request dataset. Alternatively, data augmentation techniques are available to improve attributes and size of training datasets [45].

Classification of lesions becomes challenging when physical representations of different pathologes manifest extremely similarly. For example, there are several similarities between melanoma and melanocytic nevi, which poses significant challenges for differentiation between the two [46]. Malibari et al. [46] demonstrated the effectiveness of the Improved Whale Optimization Algorithm (IWOA) with deep neural network (DNN) models in skin cancer detection and classification. The IWOA is used in this process to choose the DNN settings effectively. With a maximum accuracy of 99.90%, the comparative analysis findings demonstrated the excellent performance of the proposed optimal deep neural network (ODNN) single-bond CADSCC (Computer Aided Diagnosis model for Skin Cancer Detection and Classification) model over other previous techniques. They report that the ODNN–CADSCC model includes a number of processes, including DNN classification, U-Net segmentation, SqueezeNet-based feature extraction, Wiener filtering (WF)-based preprocessing, and IWOA-based parameter optimization. The WF-based preprocessing technique is the main method used by the ODNN–CADSCC model to eliminate noise from the dataset. In addition, the U-Net segmentation method is used to identify questionable lesions in dermoscopic images of skin cancer samples. The SqueezeNet model is utilized to produce an array of feature vectors. Finally, an IWOA model combined with a DNN model is used for picture classification.

The absence of transparent decision-making in AI-based computer-aided diagnosis (CAD) systems is a major obstacle to their successful integration into routine clinical procedures. While widely used eXplainable AI (XAI) techniques offer insights into these essentially opaque algorithms, these explanations are typically complex and difficult to understand. Given the inherent ambiguity of the description of the underlying medical condition, choices about the malignancy of skin lesions on the basis of dermoscopic images require further clarity in their explanation. To support these predictions, different studies have developed AI frameworks for biomedical image analysis that offer multimodal concept-based explanations in the form of simple-to-read verbal explanations and visual maps [46–51].

Kulkarni [52] presented a pathology-based computational method using a DNN architecture for disease-specific survival prediction. In contrast to existing approaches that use genetic markers, this novel method uses digital pictures, which eliminates the requirements for sample transportation and modification, and thus is an affordable and accessible advancement.

Another characterisation study investigated the effects of arsenic on skin [21]. Arsenic is a highly poisonous molecule present in the environment (air pollution), with long-term exposure to low quantities through food and water resulting in skin sores and cancer. This encouraged researchers to use AI to establish a remedy and comprehend the effects of arsenic through the study of skin photos, with half depicting individuals with symptoms of intoxication and the other half showing mixed images of skin unaffected by arsenic. The ultimate goal of this approach was to identify symptoms of arsenic intoxication faster and treat patients more accurately. Moreover, scientists, physicians, and other experts may be interested in this dataset, such as machine learning researchers with an interest in training AI models to distinguish between conditions resembling arsenic contamination from other diseases.

The development of characterization methods that identify affected against healthy image examples for a specific disease demonstrates a significant milestone. With some pathologically unique conditions manifesting similarly to the observer, development of models that can distinguish not only health from disease, but different diseased skin conditions from each other is the next significant step for global health initiatives in the context of skin. Examples of such categorization of pathological skin markers are further explored in this review.

This dataset provided by Emu et al. [21], demonstrates an example dataset that is well suited for the training of AI models that identify skin lesions induced by arsenic exposure. Work has already been done to extend this to other skin conditions, such as skin cancer, which is one of the greatest global healthcare burdens. Skin cancer, including both nonmelanoma skin cancer (NMSC) and malignant melanoma, is a frequent malignancy in populations with European ancestry and is becoming more prevalent [33]. According to the US Skin Cancer Foundation, more Americans are affected by skin cancer annually than by combined diagnoses of all other types of cancer [34]. Given the high risk of metastasis in skin cancer patients, early detection and affordable and accessible care are considered serious health threats.

Another classification study was carried out on the basis of photos of burned skin by Yıldız et al. [81]. They aimed to find an autonomous classifier that can exploit an artificial intelligence-based mobile application to assess burn severity from real-time images of skin burns. To this end, the application segments the burn image region and classifies the burns as first-, second-, or third-degree burns. A smartphone application named “Burn Wound Detection” was created to identify burn photos for the first time in Türkiye by this group [81].

According to the still scarce published evidence, models improve specialist care accuracy when paired with human doctors [53] albeit with a caveat concerning equality of care, due to an exacerbation of racial bias. Indeed that human-machine interaction should be carefully thought and designed in the delivery of augmented care is reinforced by several experiences [54]. Available evidences are still largely indecisive, as the same models can produce very positive results in test settings they have been explicitly designed for, whilst resulting significantly inferior in settings that have more general designs [55], although a thought should be spared for the very limited size of the experience. It is worth stressing how benchmarking in healthcare is not as straightforward as in other engineering settings, due to the messy nature of human interactions and trajectories bringing to an episode of care, and even demonstrating the superiority of specialist vs general practitioners performances has proven tricky [56]. More research should thus focus on fair and meaningful performance testing in healthcare, engaging with the medical and patient communities to better understand how bias and randomness emerge in the episode of care.

### Skin wound healing

An important aspect when defining the elements of biological phenomena that a mathematical model should encompass pertains to the primary guiding principles that the model should be as complex as necessary on the basis of (i) the research question and hypothesis to interrogate and (ii) the quantity and resolution of experimental data available to study skin mechano-biology. Furthermore, choosing the appropriate model for skin wound healing should consider the type of injury, the type of wound and the recovery phase. Although existing models of wound healing, skin tissue remodelling, and angiogenesis in the dermis rely on certain simplifying assumptions, the specific structure of the resulting model can differ significantly. There have been several excellent reviews on mathematical and computational models dedicated to wound healing [57–59]. However, a concise overview of the in silico modelling works for skin wound healing that have been developed over the last 15 years is presented below.

The pioneering paper of Flynn [60] presented a unique FE model of wound closure for simulating the incision, excision and closure of skin flaps. They modelled different excision shapes (i.e., circular, elliptical, fusiform and lazy S-plasty cuts that are relevant to skin plastic surgery) to numerically predict the surrounding stress field, the closure force along the sutured edge and the size of any extrusions. Their work demonstrated the potential of mechanistic FE models such that they can be explored to determine the optimum excision shape that minimizes the risk of skin scarring after skin closure and suturing.

Similarly, Capek et al. [61] developed a plane-stress model to evaluate the forces required for suturing selected skin wounds (specifically elliptical shapes). They performed a numerical analysis via FEs and in vivo experiments to estimate the forces induced during epidermal wound closure of elliptical wounds of varying sizes, both with and without a skin pre-stress effect. The major contribution of their work was that the amount of force required to close a wound firmly was strongly correlated with the size of the elliptical wound and skin pre-stress. Extending from the aforementioned 2D models, Chanda and Unnikrishnan [62] developed a 3D two-layered computational model of the skin (epidermis and dermis) that considered a diamond-shaped wound with a varying cross-section while also placing numerical sutures to simulate wound closure effectively. In contrast to the viscoelastic model of Capek [61], Chanda and Unnikrishnan modelled the skin as a hyperelastic material and explicitly incorporated skin prestressing. Their contribution was primarily in numerically estimating the force required for each suture to close a skin wound, which was estimated at approximately 5 Newtons with a maximum value at the centrE of the wound, where the suture was observed to require an approximately fourfold greater pull force than the first end suture. The work of Chanda and Unnikrishnan is promising in that, given the geometrical features of a wound (depth, opening), the biomechanical properties of the skin (elasticity and pre-stress) and the suture properties (wire material and cross-sectional area), a computational model can be used to estimate the force requirements of a wound suture.

Valero et al. [63] presented a continuum-based FE model of epidermal wound healing that included major biological (blood vessels and fibroblasts), biochemical (nutrients and growth factors) and biomechanical (cell traction forces and the extracellular matrix) factors that are pertinent to wound healing. The major novelty of this contribution, compared with previous works from the same group of investigators, concerns the coupling of healing-induced angiogenesis and contraction of the wound and nonlinear effects (large deformations) using an updated Lagrangian FE methodology. A key finding of Valero et al. was that an elliptical wound vascularizes two days earlier than a circular wound, despite both experiencing similar contraction levels.

Agent-based modelling (ABM) approaches have been a significant emerging research angle for the investigation of modelling wound-healing. McDougall et al. [64] presented a dermal wound healing ABM simulation method that included key extracellular materials (i.e., collagen and fibrin density and alignment), with the fibroblasts considered discrete units. They investigated in silico how manipulation of wound healing-related growth factors can modulate scar tissue formation and explored the effects of tissue remodelling on collagen alignment. In contrast to the established differential equation-based models of inflammation and wound healing, Ziraldo et al. [65] developed a computational modelling platform using ABM representation to facilitate the construction of tissue-realistic models appropriate for wound healing. Despite not being directly linked to skin healing, their hybrid model was designed to simulate pressure ulcer formation in spinal cord-injured and uninjured patients to identify control points that reduce stress caused by tissue ischaemia or reperfusion. A few years later, Boon et al. [66] proposed a cell-based ABM model to simulate the dynamics of tissue contraction in dermal wounds; this model was more advanced since it accounts for the ingress of leukocytes and the regeneration of collagen. Their cell-based model was capable of replicating the biological processes that take place during wound contracture while also combining with the role of the immune system during healing. The key takeaway of Boon’s work was that the amount of final contraction was positively correlated with the migration velocity of leukocytes, suggesting that the immune system functions well to prevent contractures.

In the same year, Vermolen and Gefen [67] proposed a phenomenological 2D model to simulate the mechanical forces exerted by myofibroblasts onto the extracellular matrix, as is evident in tissue remodelling and the contractures of burns. Their model was capable of recapitulating tissue contracture, i.e., the permanent contraction of tissue after wound healing, and was used to probe the interplay between chemokines and fibroblast-experienced strains and stresses. Later, Vermolen [68] formulated a Bayesian framework for estimating correlations and likelihoods from wound healing simulations to interrogate the mechanisms of tissue contraction. Their interesting modelling approach assesses the uncertainty of a hybrid in silico model. It combines a cell-based model with a continuum-based FE model for wound contraction and explores the correlations between the final contraction of a wound and key parameters such as tissue elasticity, force exerted by fibroblasts, death rate and differentiation rate of the cells involved in wound healing and angiogenesis.

Singh and Chanda [31] proposed a personalized model for clinical wound healing via an FE procedure to numerically estimate the biomechanical stresses and strains generated due to the natural tension of the skin during progressive wound healing. Motivated by the development of a simulation tool for pre-surgical planning and robotic surgery for performing wound closure operations, they simulated different stages of clotting. They investigated the effect of the biaxial natural tension of the skin on wound closure by studying a range of diabetic, venous, pressure, and ulcerative wounds. Singh and Chanda also studied the magnitude of biomechanical forces developing in skin tissue during the different phases of healing in human skin. Later, Bai and Zeng [69] developed a 3D geometric model of an epithelial monolayer sheet that considers the biomechanics of epithelial cells and the intercellular interactions between neighbouring cells at cell–cell junctions and the cell–substrate adhesions between epidermal cells and the hypodermis (substrate). The key findings of their modelling work are that the efficacy with which a skin wound closes is related to the initial gap size, as with the early work of Capek et al. [61], whereas enhancing the lamellipodial protrusion of epithelial cells was found to accelerate wound closure. Additionally, Bai and Zeng [69] reported that biomechanical stresses are concentrated at the wound edge of the epithelial monolayer, whereas the normal cellular stress dominates the shear stress at the cell–cell interface.

### Skin permeation

Understanding how substances permeate the skin is critical in fields such as pharmacology, dermatology, and materials science. In particular, it can help develop safe and effective topical drug delivery systems and skincare products [70]. By computationally simulating the complex interactions that govern the movement of substances through the skin, researchers can study and predict permeation rates, optimize drug delivery systems, and evaluate the safety of cosmetic and pharmaceutical formulations.

One widely used approach in skin permeation modelling is the use of mathematical frameworks based on Fick’s laws of diffusion. These models assume that permeation occurs as a diffusion-driven process and often represent the skin as a series of distinct layers, each with specific permeability coefficients. While such models are computationally efficient, they are limited by their reliance on simplifying assumptions that may not fully account for the heterogeneous and anisotropic nature of the skin.

More advanced models, such as molecular dynamics (MD) simulations and machine learning-based approaches, can overcome these limitations. MD simulations provide a detailed, atomistic perspective on how permeants interact with skin lipids and proteins, enabling the exploration of mechanisms at the nanoscale. On the other hand, machine learning models leverage large datasets from experimental permeation studies to identify patterns and make predictions. These data-driven models are particularly useful in situations where traditional mechanistic models fail or where empirical data are scarce. Notably, combining different approaches to leverage their individual advantages is possible. Zhang et al. [71] presented a computational workflow that integrates MD modelling, statistical methods named quantitative structure–property relations (QSPRs), and quantum chemistry calculations. They demonstrate good accordance with multiple in vitro experimental datasets and provide open-source software to implement their approach. Other recent studies focused more on the data-driven side of computational modelling and demonstrated high accuracies with respect to skin permeability prediction [72, 73]. Overall, the field has reached a state where permeability predictions are often accurate, and their usage for clinical applications needs to be corroborated. Close collaboration with the pharmaceutical industry has the potential to play a seminal role in this context.

Notably, challenges similar to those faced by other computational skin models remain. These include accounting for inter-individual variability in skin properties such as age, hydration levels, and disease states. Additionally, the dynamic nature of the skin, including processes such as metabolism and active transport, makes it difficult to create comprehensive and universally applicable models. Moreover, modelling and predicting the permeation of product formulations comprising mixtures of different substances is problematic, as these mixtures may entail complex interactions across ingredients.

Despite these challenges, advancements in computational power, data availability, and algorithmic sophistication continue to increase the accuracy and utility of skin permeation models. As these tools evolve, they hold the promise of accelerating the development of safer, more effective trans-dermal therapies and improving the understanding of the role of the skin as a barrier and interface with the external environment.

### The ageing skin

Research into skin ageing has gained significant traction in recent years. The skin is an organ where ageing is easily visible [74]. A person’s face may provide considerable non-verbal information, including gender, age, emotion, etc. [20, 75–77]. Such information is also detectable in pictures of human faces. Numerous methods have been created by researchers to extract this type of information from facial photos for use in a variety of computer vision applications. In addition, the effects of genetic and environmental factors can be evaluated with various computational techniques to provide further understanding of skin ageing processes.

In a recent study by Want et al. [78], researchers conducted a thorough examination of the transcriptional regulatory landscape of canine skin ageing. Weighted gene co-expression network analysis (WGCNA) is a useful bioinformatic tool used to identify correlations and magnitudes of these correlations between gene expression levels to identify patterns in which genes are expressed together. This study identifies gene interactions in mechanistic pathways and gene modules associated with ageing. Alterations in the expression of these co-expressed genes were verified in aged human skin via single-cell RNA sequencing (scRNA-seq) analysis. Notably, the cell types with the greatest differential gene expression throughout ageing are fibroblasts, mitotic cells, spinous cells, and basal cells.

Combining techniques of computational gene regulatory network (GRN) construction with WGCNA, Wang et al. [78] identified target genes by examining the frequency of expression of transcription factor (TF) binding motifs. By comparing highly enriched TFs inside the computational GRNs to genes with high degrees of connectivity via WGCNA, the genes CTCF and RAD21 were identified as key regulators in ageing skin, which were further correlated with shifts in gene expression patterns associated with age-dependent blood parameters. An approach to categorize human facial photos into four age groups via ANNs and facial skin ageing characteristics was proposed by Jagtap et al. [79]. Wrinkle analysis and the Local Gabor Binary Pattern histogram (LGBPH) were used to extract the ageing aspects of facial skin. LGBPH works on the basis of applying local binary patterns, which is a method of describing image texture on the basis of spatial patterns of pixels, onto Gabor filters, which analyse the direction frequency for texture analysis. The results from LGBPH were then used to identify wrinkles and calculate wrinkle density for wrinkle analysis. Varying ratios of the LGBPH results were then used to train feed-forward neural networks back propagation, which are ANNs where the information is processed in one direction but sends feedback about error margins to adjust the weights for the next iteration, improving the way the algorithm learns to the characteristics of agEing. Using facial images from the PAL facial database, the suggested age classification framework was evaluated and trained. The results demonstrate a significant improvement in age classification accuracy, reaching 94.17% and 93.75% for males and females, respectively [79]. The mechanical characteristics were also related to wrinkles with ageing.

The potential of physics-based skin modelling for combating skin ageing, diseases and trauma has been previously reviewed [14]. This review highlights that the physical mechanisms underlying experimental observations can be elucidated by analytical models of skin wrinkles, but these models soon become overly restrictive when investigating naturally complex biological structures, non-uniform loading conditions, or more complex non-linear materials. Selected distinguished modelling works about the skin are outlined in Table 2.

**Table 2:** Computational models related to skin wrinkles

Method or computational model	Target and result of modelling	Ref
3D agent-based model of stable epidermal structure with well-organized layers	Stability of layered structure depends on cell supply rate from the basal layer. Also explores dermal stiffness effects, barrier function, and corn (clavus) formation	[23]
Experimentally based computational model of compression-induced macroscopic skin wrinkles	Wrinkle depth increases significantly under 20–30% strain; number of layers and prestress influence wrinkle formation	[24]
Experimental apparatus to analyse morphology of abrupt wrinkle shift	Around age 33, skin wrinkling pace increases rapidly, shifting appearance from stratum corneum to epidermal wrinkles	[25]
Finite-element analyses on microrelief and etched wrinkles	Fine microrelief helps prevent deep creases (deeper than 0.35 mm)	[26]
Finite strain parametric finite element model of skin	Microrelief is the dominant factor in wrinkle characteristics with modest elastic modulus ratios	[27]
3D finite element models with 2–6 anatomically motivated layers	Simulates wrinkle formations and evaluates age-related effects on dynamic surface wrinkles	[17]
Wrinkle detection using skeleton lines and novel loss function	Improves subtle wrinkle detection; shares a dataset of 1021 Chinese face images to support research	[28]
Machine learning implementation of the Baumann Skin Type Indicator (BSTI) using InceptionV3	Achieves 84% classification accuracy for skin types (e.g., wrinkle/tight, pigmented, dry/oily, sensitive/resistant)	[29]
Wrinkle Oriented Active Appearance Model (WOAAM)	Encodes wrinkle form/texture into interpretable vectors; uses KDE to generate age-representative wrinkle patterns	[30]
Finite element method for wound geometry simulation	Simulates wound healing stress/strain in various wound types to support surgical and robotic planning	[31]
Biofidelic skin phantom with DIC testing and FE modeling	Estimates mechanical response of elliptical wounds, guiding safe closure techniques in surgery/robotics	[32]

In particular, wrinkled skin sits at the crossroad of interest between dermatopathology, healthy ageing/longevity research, and cosmetics, their appeal attested by the variety of novel technologies available on the market with the promise of delay or even reversal of the wrinkling process [15]. However, most research in the field is performed on animal models, realistic in vitro models being scarcely available and under active development [16] as a consequence of the complexity of the underlying biology and physics. When developing computational models of wrinkling skin, it must be taken into account that the number of structural layers can vary from 2 to 6 depending on the district of interest, the biophysics of forces expressed by skin muscles (and locomotor mechanics when relevant), the dermal-epidermal junction interface; the thickness of the viable epidermis, the reticular dermis, and the papillary dermis [17].

### Skin microbiota

The topic of skin microbiota is important in skin research and dermatology. The interactions between microbial organisms and the skin play an important role in human health and disease. Although relevant computational modelling work is still in its infancy, insightful studies that investigate skin microbial communities using computational modelling exist. Along those lines, the recent work of Lee et al. [100] reviews in silico skin studies and provides a comprehensive picture of the field’s state of the art. Notably, although only a relatively small number of studies is described, these cover both mechanistic approaches [101] as well as data-driven models [100, 102]. A more substantial literature exists for computational models of gut microbiota, some of which is also reviewed by Lee et al..

The study of Jaiswal et al. [103] provides a data-driven, computational framework called SkinBug that incorporates a database to model and predict metabolic activity of the skin microbiome. Their tool yields intriguing information on various aspects of metabolic variability across skin sites, highlighting the need for site-specific predictions of skin microbiomes. Moreover, the capability to predict the impact of skincare products and medical ointments on skin metabolism is very valuable for biomedical applications and understanding of toxicity.

We highlight that a wide range of computational methods have been employed to model skin microbiome data and gain insightful results. For instance, the study by Wei et al. [104] employed network-based modelling to show that there are more microbial species in moist skin sites than in sebaceous skin sites. Moreover, they show that skin microbiota are relatively well-conserved over time. Overall, their results suggest that well-established network analysis methods are effective for researching the complexity and stability of the human skin microbial community.

Given that the skin is, after the gut, the organ with the largest number and diversity of bacteria in the human body, computer models are very well-suited to address the entailed complexity. However, surprisingly little computational work has been conducted in terms of skin microbiota research. In particular, very few relevant mechanistic models exist. More interdisciplinary collaboration across domain experts and more data sharing practices will help bridge current gaps, and facilitate research that can shine light on the workings of the skin microbiome. In future, it is conceivable that more advanced models of skin microbiota will inform treatment strategies and yield personalised medicine, reflecting similar trends as in the context of the gut microbiome [105].

### In silico approaches for virtual clinical trials and digital twins in dermatology

Evidence accumulation and systematization are key bottlenecks in timely market governance, especially when dealing with highly regulated domains, such as healthcare. Thus, it comes as no surprise that players in the biomedical field would regard the acceleration and augmentation of research and innovation practices by digital means very keenly, with the first speculations on the potential impact of computer technologies in medical practice dating back to the 1960 s [86] and studies of the feasibility of internet trials dating to the early 2000 s [87]. Progress has been steady over the period, albeit not glamorous, with one of the first demonstrations of the feasibility of applying ML to medicine, the CASNET model (a consultation program for glaucoma) already in the 1970 s [88] and the Physiome (“physio” = life + “ome” = as a whole) project first presented in a report from the Commission on Bioengineering in Physiology to the International Union of Physiological Sciences (IUPS) Council at the 32nd World Congress in Glasgow in 1993. However, it is only with the growing availability of computing resources and new models of procurement thereof that the field experienced a true coming of age at the turn of the millennium.

Clinical trials are estimated to be at a value of USD 80.7 billion in 2023, with a growth projection at a compound annual growth rate (CAGR) of 6.49% from 2024 to 2030, up to USD 123.5 billion by then, with a current revenue share of 50.3% for the North American market [89]. In the case of dermatology, the market valuation for cosmetics should also be considered: the cosmetics market was valued at USD 617.20 billion in 2023 and growing at a CAGR of 8.81% from 2024–2033 [90].

This highlights the impact that randomized controlled trials (RTCs) in applications of dermatology and skin research have on the global scale, both in healthcare and socioeconomic implications. Thus, work into improving the pipeline of concept for application is imperative.

RTC augmentation practices can aid in reducing uncertainty and/or improving the generalizability of RTCs either by increasing the degree of uncertainty or by better defining the potential outcomes ahead of the study to improve the confidence of analyses and interpretations. Although commonly adopted, well explored and beyond the scope of the manuscript, here, we focus less on augmentation strategies based on data pooling and instead explore the following procedures for computational model-based augmentation: “digital twins” and “synthetic data”.

Digital twins are virtual, computer-based models of entities or systems that can be used to simulate behavioUr or/and forecast the trajectory of the physical item it represents [91]. The concept of these virtual copies is their ability to replicate substantial internal dynamics on the basis of data gathered from the actual system of interest. This enables observation of how the digital entity would respond and react in different situations. This type of synthetic data, which is obtained artificially through mathematical models and algorithms instead of real-world data, allows predictions to be made and data about the physical entity to be collected without being subjected to direct investigation [92].

Digital twins work on the premise that, while providing information, they are also frequently updated with real-time information from their physical twin. This allows for consistent parameter updates to provide the most adjusted and optimized versions of the digital twin for the best possible performance. Digital twins conceptually originated for engineering purposes, but their undeniable potential in personalized medicine has driven significant advancements in healthcare applications. The ability to model the cellular and physiological interactions of biochemical pathways provides many opportunities in healthcare fields such as disease management, drug targeting, patient monitoring and more [93].

With respect to dermatology, as early as 1997, it was estimated that a digital image could effectively substitute for a dermatological visit in more than 80% of cases [94], which further improved the use of digital methods with the advent of modern deep learning technologies [95]. This, however, comes with caveats concerning the generalizability of performance results [96], as is often the case for medical ML, because of a lack of standardization for reporting methods and benchmarks, offering a view of a biomedical domain readily amenable to acceleration and improvement of practices by computational methods.

Nevertheless, dermatology and skin biology are surprisingly untouched by efforts in the space of digital twins [97]. What is needed to establish a digital twin ecosystem capable of impacting the medical market? First, it requires open or otherwise properly governed and auditable data collection. The human skin is a complex organ with multiple layers, and structural complexity is needed to sustain immune, sensing, and repair functions. Creating models requires access to reliable data, and guaranteeing the quality of models further requires provenance, veracity, and mode of use certifications. Data repositories should include codified knowledge about structure, variability in anatomy, age, sex, clinical reports, etc.

Furthermore, when hypothesis-driven models are considered, the computational biology community should embrace standardisation practices that are widely accepted to significantly contribute to the success of ML research and deployment. As Python has been the vehicle for the generation of a competitive and accessible arena for ML, by ensuring a low entry barrier for the development of early idea prototypes and sufficient interoperability to benchmark alternative solutions against each other, digital twins ought to leave behind the heroic practice of developing small models from scratch and should embrace one or more powerful model development suites that are being built and maintained by a handful of communities [12, 98].

## Discussion & outlook

Computational and mathematical models have yielded fundamental insights into many complex systems in biology, bioengineering and biomedicine. It is therefore unsurprising that researchers of the skin are increasingly employing computational tools to help better understand processes of the skin, as well as skin-related applications and pertinent biomedical topics. Here, we provide a comprehensive overview of relevant lines of work, highlighting the strengths and weaknesses of different computational methodologies.

As dermatology, and science in general, becomes more interdisciplinary and multifaceted, the depth and variety of computerized techniques will doubtlessly advance further. Nevertheless, it emerges as a bigger picture where complementary approaches allow one to address different goals. Conveying the capabilities of such methods is paramount for their future use and cross-fertilization across scientific disciplines. As we presented in the second chapter of this review two main classes of computational approaches to simulating and understanding the complex biophysics/biomechanics of the skin are available: *model-based* and *data-driven* approaches (Table 1) – these are the two extremes of a spectrum, and hybrid models can exploit both classes respectively. On the one end, the class of *model-based* (or, as some refer to them as physics-based models) approaches, they are typically grounded in continuum mechanics and thermodynamics to explicitly represent skin’s multi-layered structure (i.e., epidermis, dermis, hypodermis) and its key biological constituents, via FE modelling for example. Their primary strength is founded in their high interpretability to provide mechanistic insights and perform simulations of pertinent biological scenarios (e.g., graft surgical interventions or transdermal drug delivery) even with limited data. However, such models are attributed to be computationally intensive when simulating large or highly detailed structures of the skin, which limits their use in real-time applications; while also formulating accurate mathematical models that can capture the intricate biology of the skin requires specialized biomechanical and biological testing to inform such a model. On the other end, data-based models are primarily based using ML or DL techniques, which bypass the need to provide explicit mathematical or biophysical rules, and they learn by “reading” patterns from experimental or clinical evidence (e.g., histological data/images, cutometer readings, etc.). The literature survey presented in the sections above demonstrates that data-based models can identify complex, often non-intuitive, correlations within high-dimensional biological datasets, while also permitting significant faster predictions, which makes them suitable for real-time applications (e.g., rapid disease skin diagnosis diagnosis, or computer-aided surgery. Despite of this however, data-based models are seen as “black boxes” and offer little insight into the underlying biological mechanisms of the skin. Equally important is the fact that the performance of these models is heavily constrained by the quantity and quality of the data employed for the model training, while they often struggle to generalise to biological conditions as for instance in rare skin diseases or in diverse ethnicities. Approaches that utilise different types of methods can help address context-specific issues, such as for instance the lack of experimental data on particular model parameters. Along those lines, model-based methods often utilise pre-existing background information to improve the accuracy of a given computational model. On the other hand, ML and DL can help identify patterns based on empirical data. It is therefore the task of the investigator to determine the right balance and utilise the most suitable method for any given problem in skin research. Thus, the choice of model (or models) depends on balancing accuracy, computational resources, and accomplishing specific research or clinical objectives. As mentioned above, a new alternative that is gaining traction is the use of hybrid models that combine elements of multiple models, which may fall in either of the two classes. These hybrid models can leverage the strengths of each class to better simulate the multifaceted nature of skin biomechanics, by incorporating imaging data (MRI, ultrasound, histology) into the model and permit personalised and high-fidelity simulations of the skin biophysics.

Despite the wealth of current computational techniques and knowledge, significant challenges with respect to real-world applications remain. Along those lines, the skin is a highly personal organ, and it is important to avoid data biases in terms of age, race or sex. Failure to avoid such biases will necessarily lead to biases in computational methods as well, which can lead to misdiagnosis and potentially dangerous medical advice. Hence, any biomedically relevant computational dermatology approach should aim for personalised parametrisation. Ideally, non-invasive, cost-effective and quick methods to measure and assess relevant properties are needed. Moreover, given the complex network of interactions in the body, many data modalities are likely important for optimizing medical impact. Among these, omics data, lifestyle-related data and even weather-related information can, in principle, play a role in determining the current and likely future state of the skin.

## Future perspective

In the future, we hope and expect the gap between theoretical and experimental dermatology to narrow, so that methodological challenges can be reduced and hypotheses, including their implications, can be thoroughly assessed. A core requisite for such increased interdisciplinary cooperation resides in the implementation of user-friendly and adaptable software. Along those lines, open-source software plays a crucial role. We highlight the need for cross-disciplinary interactions and the exchange of ideas, which enables truly user-friendly software. The reason is that only when users are directly involved in the development of the software are necessary features and customizable functionalities appropriately acknowledged, supported and realized. We have demonstrated relevant activities and outputs [12] and aim to continue working toward a future with stronger interdisciplinary, cost-efficient and ethical toolsets.

In addition to software, more widespread sharing and use of common datasets is crucial. In other areas of computational biology, such as computational neuroscience, large amounts of neuroimaging data or electrical activity recordings can be made freely available [99]. This availability not only enables more scientists to conduct research but also serves the purposes of reproducibility, benchmarking, and standardisation. Furthermore, sharing these data and resources on a broader scale can ensure that dermatological research remains dynamic, open, and ever-evolving. This will help address pressing challenges in the field, including improving diagnostic accuracy, personalizing treatments, and developing cost-effective, ethical solutions to skin-related health problems. In summary, the future of dermatology can be shaped by a combination of advanced computational models, collaborative software development, and a shared commitment to open data, ultimately contributing to more efficient, equitable, and effective scientific progress.

## Data Availability

No datasets were generated or analysed during the current study.
